# Googling DNA sequences on the World Wide Web

**DOI:** 10.1186/1471-2105-10-S14-S4

**Published:** 2009-11-10

**Authors:** Mehrdad Hajibabaei, Gregory AC Singer

**Affiliations:** 1 Biodiversity Institute of Ontario, Department of Integrative Biology, University of Guelph, Guelph, Ontario, N1G 2W1, Canada

## Abstract

**Background:**

New web-based technologies provide an excellent opportunity for sharing and accessing information and using web as a platform for interaction and collaboration. Although several specialized tools are available for analyzing DNA sequence information, conventional web-based tools have not been utilized for bioinformatics applications. We have developed a novel algorithm and implemented it for searching species-specific genomic sequences, DNA barcodes, by using popular web-based methods such as Google.

**Results:**

We developed an alignment independent character based algorithm based on dividing a sequence library (DNA barcodes) and query sequence to words. The actual search is conducted by conventional search tools such as freely available Google Desktop Search. We implemented our algorithm in two exemplar packages. We developed pre and post-processing software to provide customized input and output services, respectively. Our analysis of all publicly available DNA barcode sequences shows a high accuracy as well as rapid results.

**Conclusion:**

Our method makes use of conventional web-based technologies for specialized genetic data. It provides a robust and efficient solution for sequence search on the web. The integration of our search method for large-scale sequence libraries such as DNA barcodes provides an excellent web-based tool for accessing this information and linking it to other available categories of information on the web.

## Background

The post genomic era presents us with an ever increasing amount of DNA sequence and sequence-related data. Bioinformatics platforms such as those of National Center for Biotechnology Information (NCBI) provide suites of sequence search and analysis tools. However, new web-based technologies can significantly increase the possibilities for sharing and using sequence data in different contexts. Here we present an approach that utilizes the capabilities of conventional web-based search engines such as Google for exploring sequence and related information across multiple data sources. We have utilized this approach for sequence searches involving DNA barcodes, which are short genomic regions used in biodiversity, ecologic, and taxonomic studies for species-level identification [1,2].

In order to facilitate the use of a search engine such as Google on sequence data, we developed a character-based algorithm for DNA sequences, similar to the method recently employed by [3]. Essentially, we convert the DNA sequence into a series of "characters" that can be used to create dichotomous keys for identification. This set of characters is then compared to a library of known DNA sequences (DNA barcodes) that have, themselves, been subdivided in a similar way. Since both the query sequence and the library of sequences have been separated into short "words", we can exploit a variety of custom-built and existing word search algorithms, such as Google, to perform these searches. Here we provide a brief overview of this approach and two implementations using DNA barcoding data as an example.

## Results

### Method

We gathered all the cytochrome c oxidase 1 (CO1, *cox1*) sequences identified by the keyword BARCODE in GenBank and compiled them in a database broken into words. We also assembled all the fungal Internal Transcribed Spacer (ITS) sequences that have been generated from representative species of fungi for reconstructing fungal tree of life (AFTOL) [4]. Our user interface is composed of a simple "one box" search window. The user submits a query sequence and the program filters out gaps and breaks the sequence into words that will be piped to a conventional search engine. We have used the freely available Google Desktop Search (GDS) engine [5] for searching the sequences broken to words (but it is also possible to use the commercially available Google search appliances or any other search engine for this purpose). Using a conventional desktop computer as our hardware, a 650 base of 5' region of cytochrome *c *oxidase I gene (CO1-barcode) as query, and all the ~15,000 CO1 sequences in GenBank as our database, the search usually takes 1-2 seconds on a typical high speed Internet connection. The answer contains the species name and the sequences of 50 closest matches to the query sorted by their levels of character (word) similarity to the query, with the differences in sequence shaded (see below).

We also assembled all the fungal ITS sequences that have been generated from representative species of fungi for reconstructing fungal tree of life (AFTOL) [4]. These sequences provide an example for non-coding DNA barcodes (which are difficult to analyze using conventional alignment-based methods). ITS sequences were treated similarly as CO1 sequences. Interestingly, our method was able to provide accurate searches for these non-coding ITS sequences.

### Algorithm

There are two components to our algorithm: (1) the creation of the barcode database; and (2) the querying of that database.

#### Creation of the database

1) Barcode sequences, stored in a standard sequence format (i.e. FASTA, PHYLIP, MEGA), are read in, converted to upper-case characters, and "cleaned" to remove alignment characters or flanking ambiguous characters (poly-N's)

Example:

NNNNagcGCG---cgGATNNN → AGCGCGCGGAT

2) Each sequence is broken up into "words" consisting of a fixed number of characters, starting at the first base. If the final word is incomplete, it is deleted.

Example (for 5-base words):

GTATCGGTAACGAACTT → GTATC GGTAA CGAAC TT

3) The species and their associated words are stored in a searchable database. This database can be a custom-built hash table, or one can utilize existing search engines such as the Google Desktop Search, Google Enterprise Solutions (Google Mini or Google Appliance), Apple's Spotlight, or Microsoft's Indexing Service.

#### Querying the database

1) The user submits a sequence from an unknown specimen

2) The sequence is converted to upper-case and "cleaned" of non-DNA characters--the same operations performed on the sequences in the database

Example:

NNNN??agcgcg---CGGATNNN → AGCGCGCGGAT

3) The sequence is broken up into words in all possible "frames"

Example:

Frame 1: GTATCGGTAACGAACTT → GTATC GGTAA CGAAC TT

Frame 2: GTATCGGTAACGAACTT → G TATCG GTAAC GAACT T

Frame 3: GTATCGGTAACGAACTT → GT ATCGG TAACG AACTT

Frame 4: GTATCGGTAACGAACTT → GTA TCGGT AACGA ACTT

Frame 5: GTATCGGTAACGAACTT → GTAT CGGTA ACGAA CTT

**Figure 1:**
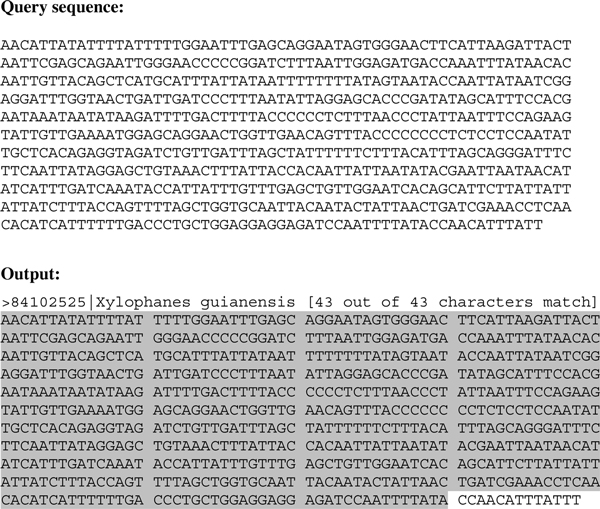
**Example Google Gene query**. The query sequence is broken into 15-base words which are then matched to a target sequence in the database. Perfectly-matching words are highlighted in the result.

4) Each set of words is queried against the database in succession. A score is assigned to each database match based on the number of words in the query sequence that exactly match words in the target sequence. The frame that returns the target sequence with the best score is considered correct.

5) For each target sequence, the species name is returned to the user, along with its full sequence with the words from the query sequence highlighted (see Figure 1)

Notes

1) Creation of the database does not require time-consuming alignments

2) The query sequence can be in any "frame" relative to the sequences in the database and they will still be matched properly

3) The order of the words is immaterial--scores are based on the number of words that match, not the order in which they match--so highly fragmented sequences will still match properly. It is possible that taking the order of words into account might improve accuracy, but this is a fundamental limitation of the underlying search algorithms.

### Implementation

#### Google Gene

We have written a custom Indexing plug-in for the Google Desktop Search (GDS) that allows the GDS to recognize common nucleotide sequence formatted files (i.e. FASTA, PHYLIP, and MEGA), read the species names, break the sequences into words, and store the results in the GDS Index. Custom-written software performs most of the steps outlined in the "Querying the Database" section of the *Algorithm *section above, but the GDS Query API is used to perform the individual searches described in Step 4. Our algorithm is highly neutral to the particular search engine used to perform the queries themselves: besides the changing search APIs, the same approach could be applied to Apple's Spotlight search (included in Mac OS X Tiger), Microsoft's Indexing Service (included in Windows Vista), or any number of alternatives.

Our current database houses over 10,000 publicly available sequences, and is set up to automatically download new barcode sequences from GenBank on a monthly basis. This system uses the NCBI eQuery API to find sequences that have been uploaded in the past 30 days that have the "BARCODE" keyword. The sequences are then downloaded, processed, and added to the Google Desktop database. This implementation of our approach is available for public use on a web server [6].

#### Tuning word sizes

**Figure 2:**
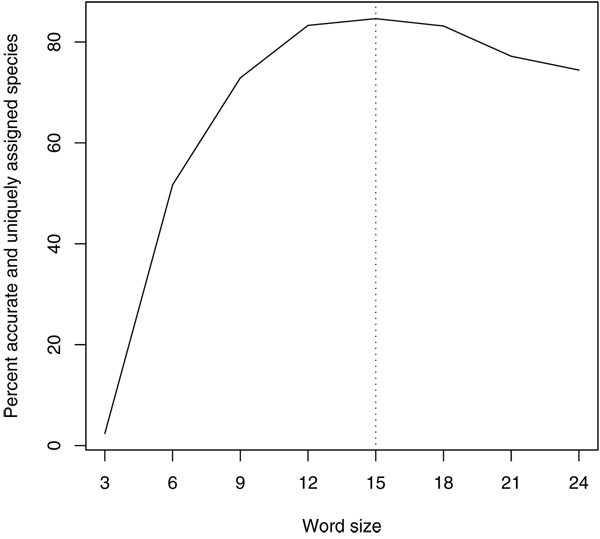
**Optimizing word sizes for DNA barcode search**. Words that are 15 bases in length (corresponding to five codons) provide a maximum degree of species assignment accuracy in our dataset.

Our algorithm is highly flexible with respect to the size of the "words" that the sequences are broken into. However, some tuning of word size is necessary to obtain optimal results. If words are too short, they will be present in every sequence and will be uninformative. On the other hand, if words are too long then small, single-nucleotide variations between sequences from the same species will reduce the overall match score as much as great differences between sequences. Speed is also a consideration: longer words allow for quicker searches. Our choice of a 15-base word size is not arbitrary, but is instead the result of a series of experiments to judge species assignment accuracy at different word lengths. We analyzed a barcode library of butterflies, moths, caddisflies, stoneflies, and mayflies and performed word-based searches of this library using word lengths ranging from 3 to 24 bases in length (in steps of three, corresponding to whole codons). As shown in Figure 2, if the words are too large then they become sensitive to small haplotype differences within species and unique assignment cannot be made robustly. Conversely, word sizes that are too small lack specificity. Our results indicate that 15-base words (five codons) provide a high degree of accuracy while maintaining many of the advantages of a longer word size. We cannot claim that this is an optimal word size for every barcode (ITS in fungi, for example), but our results indicate that for CO1 at least, accuracy is roughly constant over quite a wide range of word sizes and there is no reason to suspect that CO1 is unique in this way. Therefore, even if the optimal word size for ITS is slightly shorter or larger than 15 bases, the accuracy of species assignment should not be impacted greatly.

## Discussion and conclusion

The approach presented here, which is developed cheaply by using freely available web-based tools and conventional hardware, represents how a popular technology such as Google can be exploited for specialized genetic information. In the Barcode of Life frame work, which aims at developing a standard web-based DNA barcode library for all species, our approach provides a platform independent, fast, and accurate search for DNA barcodes. This capability is a critical element of a robust automated species identification system. In addition to searching DNA barcode data in specialized sequence data bases such as GenBank or BOLD our method also allows exploring other sources of sequence information on the web or the users' own private datasets. The user only needs an internet connection and a browser (even on a cell phone or PDA) to make use of sequence information.

## Software availability

The method described here is available in a publicly-accessible web server: . Updates and further development of the approach will be posted on this website. An additional implementation that uses the Google Mini appliance rather than the Google Desktop Search is available at the DNA Barcode Linker website .

## Competing interests

The authors have no competing interests to declare. Although intellectual property protection was initiated for this algorithm, it was not and will not be further pursued.

## Authors' contributions

MH co-developed the idea, designed the project, aided bioinformatics analysis and wrote the manuscript. GACS designed and conducted bioinformatics analysis, wrote the code and edited the manuscript. All authors have read and approved the final manuscript.

## References

[B1] Hajibabaei M, Singer GAC, Hebert PDN, Hickey DA (2007). DNA barcoding: how it complements taxonomy, molecular phylogenetics and population genetics. Trends in Genetics.

[B2] Hebert PDN, Cywinska A, Ball SL, deWaard JR (2003). Biological identifications through DNA barcodes. Proceedings of the Royal Society of London B Biological Sciences.

[B3] Sims GE, Jun SR, Wu GA, Kim SH (2009). Alignment-free genome comparison with feature frequency profiles (FFP) and optimal resolutions. Proc Natl Acad Sci USA.

[B4] James TY, Kauff F, Schoch CL, Matheny PB, Hofstetter V, Cox CJ, Celio G, Gueidan C, Fraker E, Miadlikowska J (2006). Reconstructing the early evolution of Fungi using a six-gene phylogeny. Nature.

[B5] Google Desktop Search. http://desktop.google.com/.

[B6] Google Gene Web Server. http://www.ibarcode.org/gg.

